# Targeting BRAF V600E in metastatic colorectal cancer: where are we today?

**DOI:** 10.3332/ecancer.2022.1489

**Published:** 2022-12-15

**Authors:** Rodrigo Motta Guerrero, Veronica Arnao Labajos, Sophia Lozano Ballena, Carlos Aliaga Macha, Miguel Sotelo Lezama, Cristian Pacheco Roman, Paola Montenegro Beltran, Alejandro Figueroa Torrejon

**Affiliations:** 1 Instituto Nacional de Enfermedades Neoplásicas, Surquillo 15038, Peru; 2 Hospital Almanzor Aguinaga Asenjo, Chiclayo 14001, Peru; 3 Centro Oncológico ALIADA, San Isidro 15036, Peru; a https://orcid.org/0000-0002-8086-3513; b https://orcid.org/0000-0001-7079-1010; c https://orcid.org/0000-0002-7868-6802; d https://orcid.org/0000-0003-0237-7058; e https://orcid.org/0000-0002-8861-9355; f https://orcid.org/0000-0003-2359-5126; g https://orcid.org/0000-0002-1484-9537; h https://orcid.org/0000-0001-8466-0097

**Keywords:** colorectal neoplasms, antineoplastic agents, drug therapy, immunotherapy

## Abstract

Colorectal cancer (CRC) is the second most frequent cause of direct cancer death worldwide. The study of the molecular state of oncogenes has predictive and prognostic value in metastatic CRC (mCRC). The B-raf proto-oncogene (BRAF) gene mutation represents the 8%–12% of all mutations in mCRC. The *BRAF V600E mutation, *considered the most common alteration of *BRAF*, corresponds to a constitutive kinase with a high activating capacity of the RAS/RAF/MEK/ERK pathway after a cascade of successive phosphorylations in the transcription of genes. *BRAF V600E mutation* is more prevalent in women, elderly, right-sided colon cancer and Caucasian population. Unfortunately, it is considered a poor predictive and prognosis biomarker. Patients with mCRC *BRAF V600E mutated (BRAFm) *are generally associated with poor response to chemotherapy and short progression-free survival and overall survival. Recently, randomised clinical trials have studied the combination of different chemotherapy regimens with angiogenic inhibitors in mCRC *BRAFm*. In addition, new anti-BRAF and immunotherapy agents have also been studied in this population, with positive results. The objective of this review is to acknowledge the biology and molecular pathway of *BRAF,* critically analyse the clinical trials and the therapy options published until today and evaluate the options of treatment according to the patient’s clinical presentation.

## Introduction

Colorectal cancer (CRC) is one of the most frequent neoplasms diagnosed worldwide. GLOBOCAN reports the diagnosis of more than 1.9 million new CRC cases during the year 2020, representing 10% of all new cases of malignant neoplasms. In addition, CRC is the second leading cause of direct cancer death and 9.4% of all deaths from malignant neoplasms [1]. Currently, the molecular profile of all patients with metastatic CRC (mCRC) is evaluated in daily clinical practice. Mutation of the *KRAS* (40%), *BRAF* (8%–12%) and *NRAS* (5%–10%) oncogenes plays a role in carcinogenesis and has predictive and prognostic value. Microsatellite instability (MSI) is described as a hypermutability phenotype resulting from deficiency of the germline DNA mismatch repair (dMMR) system (3%–5%) and sporadic cases (10%–15%). *MSI* is considered a biomarker with predictive value of response to immunotherapy, which is why it is also being studied in mCRC. The study of the mutational status makes possible to select the best treatment option (chemotherapy, monoclonal antibodies, immunotherapy) for each patient in daily clinical practice [2–7]. The mutation value of other oncogenes such as *HER-2* (2%), *MET* (2%), *ROS-1* (0.2%–2.4%) and *NTRK* (0.2%–2.4%) is currently under evaluation [8].

Patients with *mutated BRAF* generally correspond to a population with poor prognosis. Transversion at residue 1799 (T1799A) leads to amino acid substitution from valine to glutamic acid at codon 600 (p. V600E) in exon 15 (*BRAF V600E*), resulting in a highly active constitutive kinase. *BRAF V600E* is considered the most frequent alteration, representing >90% of all *BRAF mutations* [9]. This mutation is more prevalent in patients aged above 60 years, females and Caucasians. *BRAF V600E* is associated with right-sided cancer, mucinous and poorly differentiated histology, saw-tooth architecture, high CpG island methylator phenotype (CIMP) levels and *MSI-high (MSI-h)*. Patients with *BRAF V600E mutation* tend to metastasise more frequently to the peritoneum and distant lymph nodes. This biomarker is associated with short survival (approximately 10.4 months) [10]. *BRAF non-V600E mutations* (*BRAF 594* and *BRAF 596*) are found in <2% of mCRC and still do not have a clearly defined prognostic and predictive value and constitute a subpopulation with different clinical-pathological characteristics [11]. Currently, there is great interest in studying the *BRAF pathway*, evaluating treatment with specific agents and developing new management approaches for this patient population.

### BRAF gene and protein

The BRAF protooncogene is located in chromosome 7 (7q34) and encodes the cytoplasmic protein serine/threonine kinase. It is known that all RAF proteins (ARAF, BRAF and CRAF) can phosphorylate MEK (MEK1 and MEK2). BRAF is more commonly mutated and has the strongest activation capacity of MEK. BRAF is constituted by three domains: CR1, CR2 and CR3. CR1 encompasses the main RAS-binding domain, meanwhile CR2 corresponds to the serine/threonine-enriched regulatory domain. Both are located in the N-terminus of the protein. CR3 is a serine/threonine catalytic kinase domain that is located at the C-terminal and is regulated via phosphorylation [12–15].

### The BRAF signalling pathway

**Figure 1.**
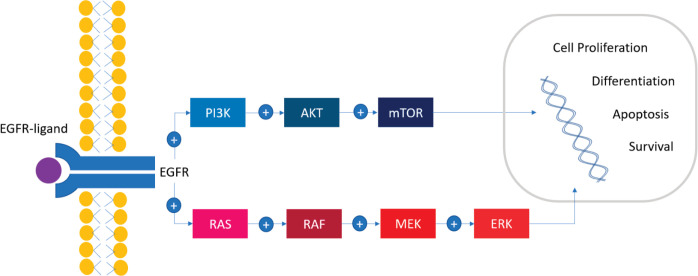
The BRAF signalling pathway. RAS/RAF/MEK/ERK signalling cascade, also known as the MAPK pathway, plays an important role in cellular proliferation, differentiation, survival and apoptosis. Multiple external sources (EGF, TGFα, epiregulin, etc.) bind to the external domain of EGFR with the consequent dimerisation of the receptor and the activation of the MAPK pathway. The activation of PI3K/AKT/mTOR pathway has been implicated as a mechanism of resistance.

A cascade of successive phosphorylations of the constituent protein molecules in the activation of the *RAS/RAF/MEK/ERK* (mitogen-activated protein kinase (*MAPK*))* pathway* regulates different mechanisms like cell proliferation, differentiation, apoptosis and survival (Figure 1). First, BRAF binds RAS-GTP, releasing the autoinhibitory CR1–CR3 interaction and cease of kinase inhibition. CR2 works as a hinge for CR1 and CR3. CR3 is a double-lobed structure that contains the kinase domain and spans the range of 457–717 amino acids. The N-terminal lobe binds ATP, while the C-terminal lobe binds substrate proteins. The two lobes flank the kinase active cleft with the active residue D576 facing into the cleft. The N-terminal lobe also contains the P-loop that stabilises ATP by electrostatic binding with phosphate group. Hydrophobic interactions also stabilise the interaction with the nucleoside ATP. After conformational changes, *KRAS* recruits and binds to cytosolic *BRAF*, forming an active homo/heterodimer with another component of the RAF family proteins. This homo/heterodimer phosphorylates and activates MEK kinases (MEK1 and MEK2) and ERK stimulating transcription factors involved in cell proliferation, differentiation, motility, apoptosis (BCL-2 regulator) and survival (via the HIPPO pathway) [12, 15].

### Mutated BRAF and carcinogenesis

The pathogenesis of CRC is a multistep mutational pathway consequence to the progressive accumulation of genetic and epigenetic alterations. Chromosomal instability and MSI are two molecular mechanisms that describe the evolution of histological alterations to carcinoma. The epigenetic hypermethylation and the inactivation of the gene MLH1 triggers the malignant development of CRC. Similarly, mutations in MMR genes (MSH2, MLH1, MSH6, PMS2 and PMS1) lead to the accumulation of mutations favouring malignant transformation. Mutation of the *Wnt signalling* is one of the initial alterations of the MSI pathway that leads to the formation of an early lesion. The following *BRAF mutation* and gene alterations, such as *IGF2R*, *TGFbR2* and *BAX mutation* allow the evolution to a late lesion and finally to carcinoma. The positive association between *BRAF V600E mutation* with *MSI-h*/*dMMR* tumours is well documented, as well as the mutual exclusivity with *KRAS mutation*. Hypermethylation is one of the molecular features of *BRAF V600E mutation* which was observed more frequently in CRC with high CIMP (77%), compared to low or negative CIMP (18% and 0%, respectively). Methylation of the CpG island in the promoter lesion ends in the silencing of tumour suppressor genes resulting in carcinogenesis. Therefore, *MSI-h/BRAF mutated* CRC occurs as a consequence of methylation of MLH1. This is why a high incidence of CpG island methylation is observed in *BRAF V600E mutated* CRC, regardless of MSI status. *BRAF mutation* is reported in sporadic CRC with hypermethylated phenotype, but not in hereditary CRC such as Lynch syndrome [12, 16].

### BRAF mutation and new molecular classifications of CRC

The CRC Subtype Consortium identified four molecular subtypes of CRC: CMS1 (MSI), CMS2 (canonical), CMS3 (metabolic) and CMS4 (mesenchymal). The incidence of *BRAF mutation* varies according to the molecular subtype. *BRAF mutation* is more frequently detected (45%) in the CMS1 cluster; meanwhile is less than 10% and 5% in the CMS3/CMS4 and CMS2 clusters, respectively. However, it has been reported that response to target therapy is heterogeneous in the molecular subgroups that harbours *BRAF V600E mutation* [12]. Two different molecular subtypes of *BRAF V600E mutations* have been distinguished: BM1 and BM2. BM2 corresponds to the 66% of all *BRAF mutated* CRC and is characterised by a dysregulation of events related to cycle checkpoints. BM2 group is enriched in metabolic processes, high levels of CDK1 and low levels of cyclin-D1. BM1 corresponds to the remaining 33% of *BRAF mutations.* BM1 is characterised by the activation of the *KRAS/AKT pathway,* alteration of *mTOR/4EBP1* and enhancement of EMT. BM1 group shows a stronger immunological profile (activation of *IL2/STAT5, IL6/JAK/STAT3 pathways, TNF-α* signalling through *NF-kB* and allograft rejection), enrichment in angiogenesis and *TGF-β*-mediated processes. Although the BM1 subtype appears to have worse prognosis, *MSI* status remains the dominant prognostic factor [12, 16, 17]. BM1 and BM2 frequency differs between each molecular subtype. The majority of patients with BM1 and BM2 were classified as CMS1 (70%) and CMS4 (17%), meanwhile few were found in CMS3 (5%) and CMS2 (2%). Interestingly, all CMS4 *BRAF mutations* are classified as BM1, while CMS1 *BRAF mutations* are distributed across BM1 and BM2 [12, 17].

### BRAF mutations and microsatellite instability in CRC

Microsatellites are repeating DNA sequences of 1–6 nucleotides in the coding and non-coding regions of the genome. The DNA mismatch repair system is responsible for correcting errors in DNA replication. *MSI-h* is a hypermutability condition resulting from DNA mismatch repair system deficiency. It is associated with high number of tumoral neoantigens and lymphocytes T infiltration. In addition, *MSI-h* tumours have high angiogenic potential and high microvascular density that can facilitate a local inflammatory response and metastasis. *MSI-h* frequency in advance stages of CRC is approximately 15%. About 3%–5% of mCRC have *MSI-h* due to a germline mutation of the mismatch repair genes (Lynch syndrome) or subsequently to a somatic inactivation of a gene (10%–15%), most commonly through hypermethylation of the MLH1 promoter region. The somatic inactivation of mismatch repair genes is strongly associated with *BRAF V600E mutation* (60%), which is practically absent in Lynch syndrome. Patients with *MSI-h* and *mutated BRAF* share similar clinicopathological characteristics such as old age, female gender, right sidedness, mucinous histology, poor cell differentiation, high-grade intratumoral lymphocytes and peritumoral lymphoid reactions. The predictive and prognostic value of *MSI-h* and *BRAF mutation* is a topic of interest in the clinical practice [7, 18].

### BRAF V600E mutated mCRC treatment strategies

Patients with *mutated BRAF V600E mCRC* tend to achieve median progression-free survival (mPFS) and overall survival (mOS) of only 4–6 and 10.4 months with conventional first-line therapies, respectively. The short survival presented in this population with poor prognosis led to the early use of aggressive therapy regimens [19]. For this reason, various treatment modalities have been evaluated in recent years. The main studies are summarised in Table 1.

### Bevacizumab + chemotherapy: triplet versus duplet

The main problem in clinical trials is the small number of *mutated BRAF* patients included. An analysis of the FIRE-3 study evaluated the impact of *KRAS* and *BRAF mutations* on the efficacy of FOLFIRI-bevacizumab versus FOLFIRI-cetuximab combinations in mCRC. Forty-eight patients with *mutated BRAF* were included, of which only 25 patients received FOLFIRI-bevacizumab. This small group achieved an overall response rate (ORR) of 40%; while the mPFS and mOS were 6.6 and 13.7 months, respectively [20]. An analysis of the Australian phase III AGITG MAX study showed that *BRAF mutation* did not have predictive value for bevacizumab-chemotherapy combination. *Mutated BRAF* patients benefit similarly to *wild-type BRAF* patients with FOLFOX-bevacizumab regimen [21]. Other antiangiogenics (ramucirumab and aflibercept) have a mechanism of action similar to bevacizumab. Despite not having substantial evidence of their clinical benefit, they could potentially share similar efficacy in *mutated BRAF* population [22].

Retrospective evidence reported that the association of a chemotherapy triplet (FOLFOXIRI) with bevacizumab improved PFS in *BRAF V600E mutated mCRC.* A prospective phase II trial evaluated FOLFOXIRI-bevacizumab combination as first-line in 25 patients with *mutated BRAF mCRC.* The study was designed to detect an increase in the PFS rate, from 45% to 80%, after 6 months of treatment. The main objective was achieved and the PFS rate was 84% after 6 months of therapy. The median PFS and OS was 11.8 and 24.1 months, meanwhile ORR achieved was high (72%) and the disease control rate (DCR) was 88%. Although the efficacy results were superior to those obtained with FOLFOX-bevacizumab, FOLFIRINOX regimen was highly toxic. The most frequently reported grade 3–4 adverse events (AEs) were: neutropenia (40%), stomatitis (20%), diarrhoea (13%), asthenia (13%) and venous thrombosis (13%) [23]. Another prospective phase II trial evaluated FOLFOXIRI-bevacizumab in a group of 57 patients with mCRC, of which 10 had *mutated BRAF* tumours. After a median follow-up of 28.8 months, no benefit was reported in mPFS (12.8 versus 13.1 months, HR = 0.89, 95% CI: 0.41–1.91) and mOS (23.8 versus 90.9 months, HR = 0.76, 95% CI: 0.26–2.21) between *mutated BRAF* and *wild-type BRAF* patients. However, the response rate obtained in the *mutated BRAF subpopulation* was higher (90% versus 75%). Grade III–IV toxicity was similar to that previously recorded. Most of the patients had neutropenia (49%), diarrhoea (14%), hypertension (11%), deep vein thrombosis (7%), stomatitis (4%) and neurotoxicity (2%) [24].

The TRIBE study was a prospective phase III trial that compared the efficacy of the combination FOLFOXIRI-bevacizumab versus FOLFIRI-bevacizumab in first-line. Of 508 included patients with mCRC, only 28 had *mutated BRAF* (12 received FOLFIRI-bevacizumab and 16 FOLFOXIRI-bevacizumab). FOLFOXIRI-bevacizumab had higher ORR (56% versus 42%; HR: 1.82, 95% CI: 0.38–8.78) and longer mPFS (7.5 versus 5.5 months; HR: 0.57, 95% CI: 0.27–1.23) and mOS (19 versus 10.7 months; HR: 0.54, 95% CI: 0.24–1.20). However, none of the differences were statistically significant, probably due to the limited number of *mutated BRAF* patients included [25]. Subsequently, the TRIBE-2 trial studied the FOLFOXIRI-bevacizumab combination versus FOLFOX-bevacizumab in first-line with 5FU-bevacizumab as maintenance therapy. At progression, FOLFOXIRI-bevacizumab reintroduction and FOLFIRI-bevacizumab sequence were indicated, respectively. The primary endpoint was PFS to second-line (PFS2). Six hundred and seventy-nine patients were enrolled and matched into the two treatment arms and the 10% (33) of patients in each arm had *BRAF mutation* (*N* = 66). There was no statistical difference between the median PFS2 (6.2 versus 5.6 months; HR = 0.87, 95% CI: 0.73–1.04, *p* = 0.11) between both regimens. FOLFOXIRI-bevacizumab achieved longer mPFS1 (12 versus 9.8 months; HR = 0.74, 95% CI: 0.63–0.86, *p* = 0.0002) and mOS (27.4 versus 22.5 months; HR = 0.82, 95% CI: 0.68–0.98, *p* = 0.032). In contrast to the TRIBE study, no benefit was found in *mutated BRAF* patients. It is suggested that a different comparator (FOLFOX in this study) or molecular heterogeneity of *mutated BRAF tumours* could explain these discordant results [26]. Zhou *et al* [27] performed a network meta-analysis to determine the most effective regimen in mCRC, including patients with *mutated BRAF* (*N* = 54). In this subpopulation, FOLFOXIRI-bevacizumab obtained longer mPFS than that obtained with bevacizumab duplets, although it did not reach statistical difference (HR: 0.64, 95% CI: 0.36–1.15). Recently, Cremolini *et al* [28] published a systematic review comparing the association of bevacizumab with duplet or triplet of chemotherapy in mCRC, including a considerable number of *mutated BRAF patients* (*N* = 115). FOLFOXIRI-bevacizumab did not improve mOS (13.5 versus 14.5 months; HR = 1.14, 95% CI: 0.75–1.73) compared to duplet-bevacizumab. No statistical difference was reported in PFS (HR = 0.84, 95% CI: 0.56–1.25) and ORR (HR = 1.42, 95% CI: 0.68–2.97) between both regimens. However, triplet-bevacizumab significantly increased grade 3–4 toxicity: neutropenia (45.8% versus 21.5%; *p* < 0.001), febrile neutropenia (6.3 versus 3.7%; *p* < 0.001), nausea (5.5% versus 3 %; *p* < 0.001), mucositis (5.1% versus 2.9%; *p* < 0.001) and diarrhoea (17.8% versus 8.4%; *p* < 0.001) [28]. Currently, the benefit of FOLFOXIRI-bevacizumab over duplets-bevacizumab regimens is not clear. The limited number of patients included in clinical trials limits conclusions.

### Chemotherapy + EGFR inhibitors?

It is postulated that patients with *mutated BRAF V600E mCRC* are resistant to EGFR inhibition due to activation of the *MAPK pathway* secondary to the *BRAF mutation.* There is limited evidence on the clinical benefit of adding anti-EGFR therapy to chemotherapy treatment in *BRAF-mutated mCRC.* Pietrantonio [29] published a meta-analysis evaluating the addition of anti-EGFR agents to chemotherapy regimens in patients with *mutated BRAF mCRC.* Results showed no benefit in PFS (HR = 0.88, *p* = 0.33) or OS (HR = 0.91, *p* = 0.63) with EGFR inhibitors. This result reinforces the role of *mutated BRAF* as a predictor of resistance to anti-EGFR agents. However, Rowland *et al* [20] point out the lack of evidence to conclude whether EGFR inhibitors are effective in *mutated BRAF* tumours*.* An analysis of the FIRE-3 study compared FOLFIRI-cetuximab versus FOLFIRI-bevacizumab in *mutated BRAF* mCRC (*N* = 48) without finding significant differences. FOLFIRI-cetuximab and FOLFIRI-bevacizumab had similar median PFS (6.6 versus 6.6 months; HR = 0.84, 95% CI: 0.47–1.51, *p* = 0.56) and OS (12.3 versus 13.7 months; HR = 0.79, 95% CI: 0.43–1.46, *p* = 0.45). The German VOLFI study (*N* = 63) evaluated the benefit of FOLFOXIRI-panitumumab versus FOLFOXIRI in wild-type *RAS mCRC.* Only 16 patients with *mutated BRAF* were included in this trial. FOLFIRINOX-panitumumab obtained more than three times the ORR (71.4% versus 22.2%; OR: 8.75, *p* = 0.126) than the triplet without the anti-EGFR. A subsequent survival analysis determined that the study population achieved a higher mOS with panitumumab (35.7 versus 29.8 months; HR = 0.67, 95% CI: 0.41–1.11) in the cohort of patients with unresectable disease. Although there are conflicting results regarding the benefit of EGFR inhibitors, there is no robust evidence to support adding them to the combination of first-line cytotoxic agents for *mutated BRAF mCRC* [20, 29–31].

### Immunotherapy: new alternative in MSI-h/dMMR

Immunotherapy has been evaluated as an alternative treatment in mCRC in clinical trials that included patients with *BRAF mutation.* The Keynote-164 trial (*N* = 124) studied pembrolizumab, anti-PD1 agent, in previously treated *MSI-h/dMMR mCRC,* including only 23 with *mutated BRAF.* Although the small sample of *mutated BRAF* patients, the results obtained in this subpopulation were interesting. The 55% (5 patients) of cohort A (≥2 prior lines of treatment) and 20% (1 patient) of cohort B (≥1 prior line of treatment) with *BRAF mutation* achieved response with pembrolizumab. In addition, pembrolizumab was safe in the population of therapy. Only 16% of patients had grade ≥ 3 AEs: pancreatitis (3%), fatigue (3%), hepatitis (2%), pneumonitis (2%), asthenia (2%), arthralgia (2%) and skin toxicity (2%). Only the 3% of patients discontinued treatment due to toxicity [32]. The multicohort Checkmate-142 trial (*N* = 74) evaluated nivolumab, another anti-PD1 agent, in heavily pretreated *MSI-high/dMMR* mCRC*.* Only 16% (12) of patients had *mutated BRAF.* The 25% (3 patients) achieved ORR, while 75% (9 patients) achieved disease control beyond 12 weeks of therapy. Nivolumab was also well tolerated; the most frequent grade ≥ 3 AEs were elevated lipases (8%) and amylases (3%) [33]. Another of the cohorts of the Checkmate-142 trial (*N* = 119) studied the safety and efficacy of nivolumab associated with an anti-CTLA4 agent (ipilimumab). This cohort included 29 heavily pretreated patients with *MSI-h/dMMR* and *mutated BRAF* mCRC. In the *mutated BRAF* subpopulation; ORR and DCR were 55% and 79%, respectively. Nivolumab-ipilimumab was relatively more toxic than anti-PD1 monotherapy. The 20% of patients had grade ≥ 3 AEs: elevated AST (8%), elevated ALT (7%), diarrhoea (2%), fatigue (2%), pruritus (2%) and skin rash (2%). 13% of patients discontinued therapy due to toxicity [34]. Results from another Checkmate-142 cohort, presented at ASCO GI (2020), evaluated the efficacy of first-line nivolumab-ipilimumab. Of the 45 patients diagnosed with *MSI-h/dMMR* mCRC*,* only 17 patients had *BRAF mutation.* The ORR (77%) was the highest obtained to date with any treatment in *mutated BRAF* patients; however, the small number of patients included limits the knowledge of the real clinical benefit of this regimen. Apparently, the anti-PD1/anti-CTLA4 combination presented less toxicity than that reported in second line. The 16% had grade ≥ 3 AEs and only 7% discontinued treatment [35].

Immunotherapy was finally studied in a phase III trial in first-line. The Keynote-177 study compared pembrolizumab versus chemotherapy as first-line in *MSI-h/dMMR* mCRC*.* The pembrolizumab group achieved significantly longer mPFS (16.5 versus 8.2 months; HR: 0.60, 95% CI: 0.45–0.80; *p* = 0.0002) and ORR (43.8% versus 33.1%) than the patients who received chemotherapy. At the moment the results were published, the data for the survival analysis was still immature and median OS was not reached. Despite this, the study Keynote-177 meant a change in the treatment paradigm of *MSI-h/dMMR* mCRC. The 22% (34) of patients in the pembrolizumab group and 23% (34) in the chemotherapy group had *mutated BRAF.* It is remarking that benefit of PFS was reported both, in *wild*-type *BRAF* (HR: 0.50, 95% CI: 0.31–0.80) and in *mutated BRAF* (HR: 0.48, 95% CI: 0.27–0.86) patients. Though the main objective of the study was not to evaluate the efficacy of pembrolizumab in patients with mutated oncogenes, it is interesting to notice clinical benefit in this subpopulation. As described in the study Keynote-164, toxicity was low and manageable. Only 9% reported grade ≥3 AEs [36]. The last results updated were presented in the ‘Gastrointestinal Cancers Symposium’ of the year 2021. Patients who received pembrolizumab presented longer PFS with the second line of therapy (PFS2). In addition, the mOS was significantly longer with the anti-PD1 agent. The chemotherapy group reached mOS of 23.5 months, while the pembrolizumab group did not reach median OS (HR: 0.63, 95% CI: 0.45–0.88) yet [37]. A recent meta-analysis evaluated the prognostic and predictive value of the *BRAF mutation* in *MSI-h/dMMR* mCRC*.* M*utated BRAF* patients had shorter survival than *non-mutated BRAF* patients in all clinical stages (I–IV) (HR: 1.57; 95% CI: 1.23–1.99). The ORR obtained was similar among *mutated BRAF* and *wild-type BRAF* patients treated with immunotherapy (OR: 1.04, 95% CI: 0.48–2.25). According to these results, survival of *MSI-h/dMMR* mCRC with* BRAF mutation* may significantly benefit with immunotherapy [38]. During the preparation of this review, no meta-analyses evaluating the efficacy of immunotherapy on PFS or OS in *mutated BRAF/MSI-h/dMMR* mCRC were found*.* The limited number of *mutated BRAF* patients included in clinical trials does not allow the development of more complex studies. However, the results suggest a potentially robust benefit with immunotherapy in this population.

### BRAF inhibition: looking for the optimal combination

The efficacy of treatment with BRAF inhibitors was studied in small clinical trials, obtaining modest benefit in *mutated BRAF V600E* mCRC*.* A phase II trial (*N* = 10) reported that vemurafenib achieved mPFS of 2.1 and mOS of 7.7 months, without improving ORR. Another phase II trial (*N* = 21) reported similar results in survival (mPFS = 4.5 months and mOS = 9.3 months) and low ORR (5%) with vemurafenib [39, 40]. A phase I trial (*N* = 18) evaluated the efficacy of encorafenib, selective RAF kinase inhibitor, without positive results. Encorafenib achieved mPFS of 4 months, without improving ORR [41]. Preclinical studies suggest that BRAF inhibitors do not produce sustained inhibition of the *MAPK pathway,* which leads to a lack of response to these agents [42].

Laboratory studies suggest that the combined BRAF and MEK inhibition could achieve greater suppression of the *MAPK pathway* and greater antitumour efficacy. Unfortunately, the results were not the most favourable. The combination of BRAF inhibitors (dabrafenib) with MEK inhibitors (trametinib) was studied in 43 patients with heavily previously treated *mutated BRAF mCRC* (51% received ≥ 3 lines of therapy). Tumour samples were evaluated, showing lower levels of *phosphorylated ERK* compared to samples before the beginning of therapy (47%–24%). The mPFS was 3.5 months and the median duration of therapy was 3.6 months, meanwhile 23% (10 patients) continued therapy for more than 6 months. The 12% (5 patients) had partial response and 1 patient achieved complete response. Interestingly, the complete responder and two partial responders were reported to have *PI3K pathway mutations.* However, severe toxicity was significant with the combination. The 98% of patients presented AEs and 58% had grade ≥ 3 toxicity; the most frequent were: anaemia (16%), pyrexia (12%), vomiting (7%) and fatigue (7%). Pyrexia was the most common reason for discontinuing therapy (30%) [42].

Activation of the *PI3K/AKT pathway* has been identified as a mechanism of resistance to BRAF inhibitors in *mutated BRAF* mCRC*.* A recent publication reported that genetic alterations in *EGFR* and *PI3K* were associated with poor response to targeted treatment and the development of secondary resistance mutations. A phase Ib trial evaluated the safety of the encorafenib-cetuximab regimen (*N* = 26) compared with the same regimen associated with a PI3K inhibitor (alpelisib) (*N* = 28) in refractory *mutated BRAF mCRC.* The regimen including the PI3K inhibitor was well tolerated and only two patients reported dose-limiting toxicity. The combination with alpelisib achieved ORR of 18% and mPFS of 4.2 months. However, no further published results on PI3K inhibitors in *mutated BRAF mCRC* were found until the preparation of this review [43, 44].

Subsequently, preclinical studies demonstrated a decreased sensitivity to BRAF inhibitors with transient suppression of *phosphorylated ERK,* followed by the reactivation of *RAS* and *C-RAF,* mediated by the *EGFR pathway.* The combination of BRAF and EGFR inhibitors had a synergistic effect *in vitro*, resulting in sustained suppression of the *MAPK pathway* and the improvement of the efficacy of tumour growth inhibition [45]. These results led to the study of the addition of an EGFR inhibitor to the BRAF inhibition in clinical trials. A phase II trial (*n* = 27) evaluated the vemurafenib-cetuximab combination in pretreated *mutated BRAF* mCRC. Only one patient achieved partial response, but approximately half of the population had tumour shrinkage without achieving partial response. The median PFS and OS were 3.7 months (95% CI: 1.8–5.1) and 7.1 months (95% CI: 4.4–not reached), respectively [41]. Another trial evaluated the efficacy of BRAF inhibition, via anti-BRAF (dabrafenib) with anti-MEK (trametinib) agents in association with EGFR inhibition (panitumumab). One hundred and forty-two patients with previously treated *BRAF V600E mutated* mCRC (35% received ≥ 2 lines) were enrolled and distributed into three arms: dabrafenib-panitumumab, dabrafenib-panitumumab-trametinib and trametinib-panitumumab. The primary objective was to assess the ORR of the three combinations and dabrafenib-panitumumab-trametinib achieved the highest ORR (21%). Dabrafenib-panitumumab and trametinib-panitumumab had 10% and 0% of ORR, respectively. However, there was no superiority in PFS between the three arms. The mPFS was 4.2, 3.5 and 2.6 months with dabrafenib-panitumumab-trametinib, dabrafenib-panitumumab and trametinib-panitumumab combinations; respectively. OS data was still immature when the study was published; however, mOS was achieved in dabrafenib-panitumumab (13.2 months; 95% CI: 6.7–22 months) and trametinib-panitumumab (8.2 months; 95% CI: 6.7–22 months) groups. Dabrafenib-panitumumab-trametinib combination was associated with higher *MAPK suppression* (60%) compared to the dabrafenib-panitumumab (23%) and trametinib-panitumumab (41%) regimens. The addition of an EGFR inhibitor to BRAF inhibition seems to have a slight benefit in *BRAF V600E mutated* mCRC. However, the immature data do not allow establishing a clear clinical benefit in survival. We need a longer follow-up time to validate these results. The toxicity of the triplet was notoriously high, 70% presented AEs grade ≥ 3, the most frequent being: skin rash (11%), acneiform dermatitis (10%), fatigue (7%) and diarrhoea (7%) [46].

The phase III BEACON study evaluated the BRAF inhibition through the combination of a BRAF inhibitor (encorafenib) with a MEK inhibitor (binimetinib), and the inhibition of EGFR pathway (cetuximab) in previously treated with 1–2 lines *mutated BRAF V600E* mCRC. Six hundred and sixty-five patients were enrolled and randomised into three groups: the triplet group (encorafenib-binimetinib-cetuximab), the duplet group (encorafenib-cetuximab) and the control group (treatment of choice chosen by the investigator: cetuximab-irinotecan or cetuximab- FOLFIRI). The primary endpoints of the study were OS and ORR in the triplet group. The mOS (9 versus 5.4 months; HR: 0.52; 95% CI: 0.39–0.70; *p* < 0.001) and ORR (26% versus 2%; *p* < 0.001) were longer with encorafenib-binimetinib-cetuximab. The study was not designed to compare the outcomes between the triplet and duplet groups; but the OS and ORR obtained with triplet was similar to the result of encorafenib-cetuximab combination (ORR = 20%, mOS = 8.4 months). The mPFS was significantly higher in the triplet group (4.3 versus 1.5 months; HR: 0.38, 95% CI: 0.29–0.49, *p* < 0.001) and the doublet group (4.2 versus 1.5 months; HR: 0.4, 95% CI: 0.31–0.52, *p* < 0.001) compared to the control group. Regarding toxicity, 58% of patients in the triplet group, 50% in the duplet group and 61% in the control group presented grade 3–4 AEs. The most frequently reported AEs were: anaemia (11%), diarrhoea (10%), abdominal pain (6%), nausea (5%) and altered creatinine (5%). A subsequent safety analysis of the encorafenib-binimetinib-cetuximab regimen reported that five patients had dose-limiting toxicity; of which two presented retinopathies, one decreased left ventricular ejection fraction, and two infusional reactions related to cetuximab. The most commonly reported grade 3–4 AEs with the triplet were: fatigue (13%), anaemia (10%), increased creatinine phosphokinase (10%), increased AST (10%) and urinary tract infections (10%) [47]. The update of the study published in 2021 determined that the mOS was 9.3 months for the triplet group and 5.9 months for the control group (HR: 0.60, 95% CI: 0.47–0.75); meanwhile, the mOS for the duplet group was 9.3 months, also higher than the control group (HR: 0.61, 95% CI: 0.48–0.77). The ORR was 26.8% (95% CI: 21.1%–33.1%) for the triplet, 19.5% (95% CI: 14.5%–25.4%) for the duplet and 1.8% (95% CI: 0.5%–4.6%) for the control. The encorafenib-cetuximab combination improved OS, PFS and ORR similarly to the triplet, but with less toxicity, becoming the new standard of care in previously treated *mutated BRAF V600E mCRC* [48]. Subsequently, the ANCHOR study, a phase II trial, evaluated the same regimen in first-line for *mutated BRAF V600E* mCRC*.* The results of the first stage of this trial were presented at the ‘ESMO Gastrointestinal Congress’, reporting the results of 40 patients, who achieved ORR of 50% (95% CI: 33.8–66.2), with tumour reduction of 85%. However, the mPFS was only 4.9 months (95% CI: 4.4–8.1). Final results are still being waited today [49].

Recently, the combination of BRAF and EGFR inhibition in combination with chemotherapy has been studied. The SWOG-S1406 trial evaluated irinotecan-cetuximab versus irinotecan-cetuximab-vemurafenib treatment in 106 patients with *mutated BRAF V600E mCRC,* previously treated with 1–2 lines. Vemurafenib regimen achieved longer mPFS (4.2 versus 2 months; HR = 0.50, 95% CI: 0.32–0.76, *p* = 0.001) and higher ORR (17% versus 4%, *p* = 0.05) compared with irinotecan-cetuximab. A decreased frequency of circulating tumour *BRAF V600E DNA* was reported with vemurafenib (87% versus 0%, *p* < 0.001), with a poor incidence of acquired *RAS alterations* at progression. Grade 3–4 toxicity was more common in patients receiving vemurafenib; the most common were: neutropenia (30%), nausea (19%) and anaemia (13%). 22% of patients discontinued therapy due to toxicity [50].

### What about non-V600E BRAF mutated mCRC treatment?

As previously described, a small percentage (<2%) of patients harbours *BRAF non-V600E mutations*. *Mutated BRAF non-V600E* tumours are associated with younger age, lower degree of cellular differentiation, less frequency mucinous histology, microsatellite stability, left-sided disease and a less aggressive evolution of cancer than patients with *mutated BRAF V600E* or *wild-type BRAF* tumours. *BRAF non-V600E mutations* are usually associated with significantly longer survival. However, because of the low frequency of these mutations, there is no randomised prospective data about potential treatment agents in this population. Recently, studying reports suggest that BRAF non-V600E tumours may be sensitive to EGFR inhibitors. However, the small sample size does not allow to make definitive conclusions. In addition, it has been described long-term response with regorafenib in one heavily pretreated mCRC patient with *BRAF non-V600E*. This result was attributed to the regorafenib’s efficacy targeting the epithelial to mesenchymal pathway *in vitro*. Further prospective studies are needed to find potential agents for these mutations [9, 11, 51–53].

### Treatment in clinical practice

Patients with *mutated BRAF mCRC* correspond to a small population that has not been significantly represented in clinical trials of mCRC and has not been widely studied. Retrospective and prospective studies have reported a slight benefit in survival with cytotoxic treatment associated with bevacizumab in this poor prognosis population. The guide of the ‘Spanish Society of Medical Oncology’ (SEOM) and the ‘Consensus of Pan-Asian guidelines adapted by ESMO for the Management of metastatic colorectal cancer: JSMO-ESMO initiative endorsed by CSCO, KACO, MOS, SSO, and TOS’, both published in 2018, suggest the use of intense chemotherapy regimens (FOLFOXIRI) associated with bevacizumab in first-line therapy for *BRAF mutated mCRC* with good clinical status (ECOG 0–1), regardless of sidedness (right colon and left colon) (level of evidence IIB). The chemotherapy duplet associated with bevacizumab is suggested as the second option and the chemotherapy duplet without the anti-angiogenic agent as the third option. However, the ‘German evidence-based guideline for colorectal cancer’ (2019) acknowledges the positive results of the TRIBE study, but highlights that the small number of *BRAF mutated patients* included only allows for establishing treatment hypotheses [54–56]. The German guideline suggests that this population should receive the most effective treatment, such as the triplet, or be included in clinical trials (grade of recommendation B). The Japanese Society for Colorectal Cancer (JSCRC) (2019) guideline recommends the combination FOLFOXIRI-bevacizumab in the first-line treatment of *BRAF mutated* mCRC, according to the results of the TRIBE study [57]. The most recently published evidence, version 1 of the ‘National Comprehensive Cancer Network’ (NCCN) guideline (2022), recommends the FOLFOX or FOLFOXIRI regimens associated or not with an anti-angiogenic agent in patients who are candidates for receiving intense therapy (category II-A) [57]. This indication is more in line with the results of the TRIBE-2 study and the recently published meta-analyses by Zhou *et al* [27] and Cremolini *et al* [28]. Treatment with the FOLFOXIRI-bevacizumab regimen causes significant toxicity in daily clinical practice. Considering the latest publications of clinical trials and meta-analyses, it is consistent to consider the use of FOLFOXIRI-bevacizumab in selected patients (young, ECOG 0–1, no severe comorbidities) when the goal of treatment is to achieve response rate (symptomatic patients and/or extensive tumour involvement). In all other cases, the most appropriate indication is the use of duplet regimens with an angiogenic inhibitor. The combination of single chemotherapy agent with an angiogenic inhibitor is an alternative of treatment according to the patient’s clinical and molecular characteristics [58].

In daily practice, the mutational state of biomarkers (*KRAS, NRAS, BRAF, MSI)* of patients with mCRC is studied. Based on the results of the Keynote-177 study and the Checkmate-164 study, pembrolizumab and the nivolumab-ipilimumab combination are first-line treatment options for mCRC*-high MSI/dMMR.* Pembrolizumab was approved by the ‘Food and Drugs Administration’ (FDA) and by the European Medicines Agency (EMA), in June and December of the year 2020; respectively. The British guide ‘National Institute for Health and Care Excellence’ (NICE) (2021) recommends the use of pembrolizumab in untreated *MSI-h/dMMR* mCRC [59]. Similarly, the NCCN guideline (2022) suggests the use of pembrolizumab (preferred option) and nivolumab-ipilimumab as preferred first-line therapy in *MSI-h/dMMR* mCRC (both category II-A). FDA approved pembrolizumab and the combination nivolumab-ipilimumab for previously treated *MSI-h/dMMR* mCRC in May 2017 and July 2018, respectively [60]. The NCCN guideline (2022) recommends pembrolizumab (preferred) and nivolumab-ipilimumab as therapy options beyond the first line of treatment in *MSI-h/dMMR* mCRC (both with category II-A) [58]. Considering the design of the Keynote-177 trial (phase III with comparator) and the lower toxic profile with a single agent, the use of pembrolizumab as the agent of choice in this subpopulation of patients is valid. *BRAF mutated* patients without *MSI-h/dMMR* should receive combinations of duplet or triplet chemotherapy, ideally associated with an anti-angiogenic agent.

**Figure 2.**
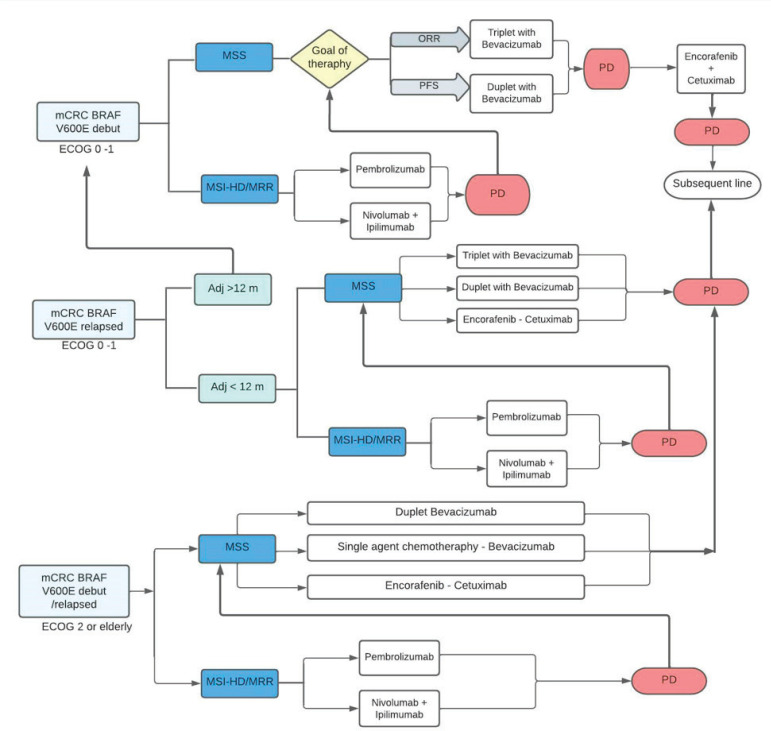
Algorithm of treatment in mutated BRAF V600E mCRC. mCRC, Metastatic colorectal cáncer; MSI-h, High microsatellite instability; dMMR, DNA mismatch repair; MSS, Microsatellite stable; ORR, Overall response rate; PFS, Progression free survival; PD, Progression of disease.

Recently, clinical trials have reported positive results with the use of regimens based on combinations of different agents. The FDA and EMA approved the encorafenib-cetuximab combination for the treatment of previously treated mCRC with *BRAF V600E mutation,* in April and June 2020, respectively. Both regulatory agencies approved the regimen based on the reported findings of the BEACON study [61, 62]. The British guide ‘National Institute for Health and Care Excellence’ (NICE) (2021) recommended the encorafenib-cetuximab combination for the treatment of adult patients with *mutated BRAF mCRC* who previously received systemic therapy. The suggested recommendation is based on the fact that the benefit provided in this population with a poor prognosis has been minimal in decades of mCRC studies and the encorafenib-cetuximab regimen is a paradigm shift in treatment. They also point out that mCRC and its treatment with conventional regimens affect the quality of life of patients. They emphasise that the toxicity of the encorafenib-cetuximab combination is manageable, which significantly improved the quality of life of the patients [63]. Similarly, the ‘National Comprehensive Cancer Network’ (NCCN) guideline (2022) recommends the combination of encorafenib (300 mg daily, PO) associated with cetuximab (400 mg/m^2^ IV, followed by 250 mg/m^2^ weekly) or panitumumab (6 mg/kg IV, every 14 days) as treatment options in patients with *mutated BRAF V600E* mCRC who progressed within 12 months of completing adjuvant chemotherapy or progressed to first-line oxaliplatin-based with/without irinotecan and with/without bevacizumab, or progressed to a subsequent irinotecan-based line (level of Evidence IIA). The encorafenib-cetuximab combination obtained interesting results in the BEACON study and is the regimen of choice after progression to first-line systemic therapy. The use of encorafenib-cetuximab could be considered when the patient progresses to adjuvant therapy with an oxaliplatin-associated fluoropyrimidine (FOLFOX/CAPOX) or relapses within 12 months of completing adjuvant FOLFOX or CAPOX, as option to treatment with FOLFIRI. Beyond the progression to encorafenib-cetuximab, a subsequent new line of therapy could be considered, according to ECOG and sequelae due to treatment toxicity. Alternatives include regorafenib or reintroduction of fluoropyrimidine therapy with or without oxaliplatin, associated or not with an anti-angiogenic agent (ramucirumab, aflibercept, reintroduction of bevacizumab) [58]. Summary in Figure 2.

### Future perspectives

Studies that associate immunotherapy agents with BRAF inhibitors are currently being carried out. As previously described, *MSI* is associated with *BRAF mutation,* secondary to epigenetic inactivation of the mismatch repair protein MSH1, for which patients are reclassified in the molecular subgroup MCS1, characterised by high hypermethylation and response to immunotherapy. The SEAMARK trial (NCT05217446) is currently evaluating the combination encorafenib-cetuximab-pembrolizumab versus pembrolizumab alone in mCRC with *IMS-h/dMMR.* However, special interest is being added to the treatment of patients with microsatellite stable (MSS). Several clinical trials are evaluating the association of BRAF and EGFR inhibition with immunotherapy in heavily pretreated mCRC patients with MSS (NCT05019534, NCT05308446, NCT04017650). In addition, the NCT04044430 study is a phase I/II trial that evaluates the safety of the combination encorafenib-binimetinib associated with nivolumab in *mutated BRAF V600E* mCRC and MSS. The NIVACOR trial (NCT04072198), single-arm phase II study, in which the treatment with FOLFOXIRI-bevacizumab-nivolumab is being evaluated in *mutated BRAF V600E* patients. The study is still in the recruitment phase. A preliminary safety analysis of the first ten patients included reported a median of 5.5 cycles of treatment [17, 64, 65].

Other new therapy strategies are the new anti-BRAF agents, such as HLX208 alone or in combination with MEK or EGFR inhibitors (NCT04965220, NCT05127759, NCT04984369). Similarly; the safety and efficacy of ERK, RAF and SHP2 inhibitors are being evaluated in phase I clinical trial (NCT04294160). Looking at preclinical studies, ‘Wee1 and ERK1/2’ are points downstream of the *BRAF pathway* in the *MAP kinase signalling cascade* and are potentially important therapeutic targets, and based on this, AZD1775 (Wee1 inhibitor) and LY3214996 (ERK1/2 inhibitor) have been tested in phase I trials. In addition, the activation of the *Wnt pathway by* the *RNF43 mutation* or* RSPO fusions* may contribute to resistance in *mutated BRAF mCRC.* Under this premise, a phase I/II trial of combined therapy of Wnt inhibitor, WNT974 (porcupine inhibitor), with BRAF inhibition is being carried out (NCT02278133) [66, 67].

Based on the positive results obtained with SWOG 1406 trial, simultaneous *EGFR* and *BRAF* inhibition associated with cytotoxic agents is effective and safe in patients with *mutated BRAF mCRC.* These results put on hold the BREAKWATER study, a phase III trial that evaluates the combination encorafenib-cetuximab associated with chemotherapy. Similarly, a phase II trial is evaluating the combination vemurafenib-cetuximab with FOLFIRI (NCT04607421, NCT03727763) [68]. Time will let us know if these results will change standard of care. Ongoing clinical trials are resulted in Table 2.

## Conclusion

Patients with *mutated BRAF V600E* mCRC harbour poor prognosis and short survival. Immunotherapy, the combination of BRAF and EGFR inhibitors and the combination of chemotherapy with angiogenic inhibitors have managed to prolong the survival of this population. Choosing the right treatment depends on each patient’s clinical and molecular features. The study of *BRAF* signalling pathway allowed the discovery of novel agents that are being studied right now. The present and future of *mutated BRAF V600E* mCRC treatment may be seen with hope.

## Conflict of interest

The authors declare that they have no financial or non-financial conflicts of interest.

## Author contributions

Rodrigo Motta Guerrero, Miguel Sotelo Lezama and Alejandro Figueroa Torrejon made the contributions to the conception and design of the work. Rodrigo Motta Guerrero, Veronica Arnao Labajos, Sophia Lozano Ballena and Carlos Aliaga Macha made the first draft. Alejandro Figueroa Torrejon, Paola Montenegro Beltran and Cristian Pacheco Roman critically reviewed the draft for intellectual content and made significant changes. All authors approved the final version of the manuscript.

## Funding statement

No funding was required for this article.

## Figures and Tables

**Table 1.** Most relevant clinical trials in mCRC with *BRAF V600E mutation*.

Trial	Design	N° of patients BRAFm	Line of treatment	Therapy	mOS	mPFS	ORR
Keynote-164	Phase II	23/124 (18.5%)	≥2 lines	Pembro	-	-	34.7%
Checkmate-142	Phase II	29/119 (24.3%)	≥2° lines	Nivo + Ipi	-	-	55%
Corcoran *et al* [46]	Phase I	142/142 (100%)	≥2 lines	Dabra + Pani Dabra + Pani + Trame Trame + Pani	13.2 months 8.2 months -	3.5 months 4.2 months 2.6 months	10% 21% 0%
BEACON	Phase III	665/665 (100%)	≥2 lines	Enco + Bini + Cetu Enco + Cetu CT + Cetu	9.3 months HR: 0.38, *p* < 0.001 9.3 months HR: 0.40, *p* < 0.001 5.9 months	4.3 months 4.2 months 1.5 months	26.8% 19.5% 1.8%
SWOG S1406	Phase II	106/106 (100%)	≥2 lines	Irino + Cetu + Vemu Irino + Cetu	- -	4.2 months 2 months HR: 0.50, *p* = 0.001	17% 4%
Checkmate-142	Phase II	17/45 (37.7%)	1 line	Nivo + Ipi	-	-	71%
Keynote-177	Phase III	57/307 (18.5%)	1 line	Pembro CT	NR 23.5 months	16.5 months 8.2 months HR: 0.60, *p* = 0.0002	43.8% 33.1%
Loupakis *et al* [23]	Phase II	25/25 (100%)	1 line	FOLFOXIRI + Beva	24.1 months	11.8 months	72%
Masi *et al* [24]	Phase II	10/57 (17.5%)	1 line	FOLFOXIRI + Beva	23.8 months HR = 0.76, NS	12.8 months HR = 0.89, NS	90%
TRIBE	Phase III	28/508 (5.5%)	1 line	FOLFOXIRI + Beva FOLFIRI + Beva	19 months 10.7 months HR = 0.54, NS	7.5 months 5.5 months HR = 0.57, NS	56% 42%
TRIBE-2	Phase III	66/679 (9.7%)	1 line	FOLFOXIRI + Beva FOLFOX + Beva	27.4 months 22.5 months HR = 0.82, *p* = 0.032	6.2 months 5.6 months HR = 0.87, NS	- -

Abbreviations: mCRC, Metastatic colorectal cáncer; N°, Number; BRAFm, BRAF mutated; Beva, Bevacizumab, Pembro, Pembrolizumab; Nivo, Nivolumab; Ipi, ipilimumab; Dabra, Dabrafenib; Vemu, Vemurafenib; Pani, Panitumumab; Trame, Trametinib, Enco, Encorafenib; Cetu, Cetuximab; Irino, Irinotecan; CT, Chemotherapy; Bini, Binimetinib; M, Months; NR, Non reach; NS, Non statistically significant

**Table 2.** Ongoing clinical trials in mutated *BRAF V600E* mCRC.

Trial	Design	Population	Line of treatment	Regimen	Objectives
NCT04965220	Phase I	Solid tumours: BRAF V600E	≥2° lines	HLX208-Trime	1° endpoint: maximum tolerated dose 2° endpoint: Cmax, ORR, AUC
NCT05127759	Phase I	mCRC: BRAF V600E	≥2° lines	HLX208	1° endpoint: ORR 2° endpoint: PFS, OS
NCT04984369	Phase II	mCRC: BRAF V600E	≥2° lines	HLX208-Cetu	1° endpoint: ORR 2° endpoint: PFS, OS
NCT04294160	Phase Ib	mCRC: BRAF V600E	≥2° lines	LTT462-Dabra versus LTT462-Dabra-Trame versus LTT462-Dabra- LXH254 versus LTT462-Dabra- TNO155 versus LTT462-Dabra-Sparta versus TNO155-Dabra-Trame versus LTT462-Dabra-Tisle	1° endpoint: DLTs 2° endpoint: BOR, PFS, ORR, DoR, DCR, Cmax
NCT02278133	Phase I/II	mCRC: BRAF V600E + RNF43m or RSPO fusions	≥2° lines	WNT974-LGX818-Cetu	1° endpoint: toxicity 2° endpoint: ORR
NCT03668431	Phase II	mCRC: BRAF V600E + KRAS/NRASwt	≥2° lines	PDR001-Dabra-Trame	1° endpoint: ORR, toxicity 2° endpoint: PFS, OS, DCR, DoR
NCT05019534	Phase I	mCRC: BRAF V600E + MSS	≥2° lines	Vemu-Cetu-Camre	1° endpoint: toxicity 2° endpoint: ORR, PFS, OS, DCR
NCT04044430	Phase I/II	mCRC: BRAF V600E + MSS	≥2° lines	Enco-Bini-Nivo	1° endpoint: toxicity 2° endpoint: ORR, PFS, OS, DoR, DCR
NCT04017650	Phase I/II	mCRC: BRAF V600E + MSS	2°–3° lines	Enco-Cetu-Nivo	1° endpoint: toxicity 2° endpoint: ORR, PFS, OS, DoR, DCR
NCT05308446	Phase II	mCRC: BRAF V600E + MSS	≥2° lines	Enco-Cetu-Nivo versus Enco-Cetu	1° endpoint: PFS 2° endpoint: ORR, OS, DoR
NCT05217446	Phase II	mCRC: BRAF V600E + MSI-h/dMMR	1° line	Enco-Cetu-Pembro versus Pembro	1° endpoint: PFS 2° endpoint: ORR, OS
NCT04072198	Phase II	mCRC: BRAF V600E + RASwt	1° line	FOLFOXIRI-Beva-Nivo	1° endpoint: ORR 2° endpoint: PFS, OS, toxicity, DoR, QoL
NCT03727763	Phase II	mCRC: BRAF V600E + RASwt	1° line	FOLFIRI-Vemu-Cetu	1° endpoint: ORR 2° endpoint: DCR, PFS, OS
NCT04607421	Phase III	mCRC: BRAF V600E	1° line	CT-Enco-Cetu versus Enco-Cetu versus CT	1° endpoint: toxicity 2° endpoint: ORR, PFS, OS, DoR, TRR, DCR

Abbreviations: mCRC, Metastatic colorectal cancer; Enco, Encorafenib; Cetu, Cetuximab; CT, Chemotherapy; Vemu, Vemurafenib; Beva, Bevacizumab; Nivo, Nivolumab; Pembro, Pembrolizumab; Bini, Binimetinib; Camre, Camrelizumab; Dabra, Dabrafenib; Trame, Trametinib; Tisle, Tislelizumab; Sparta, Spartalizumab; Trime, Trimetinib; ORR, Overall response rate; PFS, Progression free survival; OS, Overall survival; DoR, Duration of response; DCR, Disease control rate; QoL, Quality of life; BOR, Best overall response; Cmax, Maximum serum concentration; DLTs, Dose-limiting toxicity; AUC, Area under curve
